# Divergent molecular strategies of next-generation activated factor VIII mimetics: insights from a direct comparative analysis

**DOI:** 10.1016/j.rpth.2026.106906

**Published:** 2026-08-12

**Authors:** Tadashi Matsushita

**Affiliations:** Blood Service Headquarters, Japanese Red Cross Society, Minato-ku, Tokyo, Japan

## 1 Introduction

The study by Lund et al. [1] in this issue provides exceptional documentary value by performing a head-to-head comparison of all 3 mimetics—denecimig and sequence-identical analogs (SIAs) of zemocimig and emicizumab—under standardized, identical conditions. The therapeutic landscape of hemophilia A (HA) was previously transformed by emicizumab, a bispecific antibody mimicking activated factor (F)VIII (FVIIIa) function, represented a paradigm shift in the prophylaxis of HA [[2], [3], [4]]. By bridging activated FIX and FX, emicizumab effectively bypasses the need for FVIII, significantly reducing the treatment burden through subcutaneous administration. However, the quest for “nonhemophilic” levels of hemostatic protection has driven the development of next-generation mimetics, including denecimig (Mim8) [[5], [6], [7], [8], [9], [10]] and zemocimig (NXT007) [[11], [12], [13], [14], [15], [16]].

A significant challenge in evaluating these newer agents is the heterogeneity of experimental conditions across different studies, which makes direct comparisons difficult.

## 2 The Core Findings: Distinct Biochemical Engines

The most significant contribution of this research [1] is the elucidation of the sophisticated biochemical mechanisms driving these 3 antibodies, particularly for zemocimig, which had not been previously characterized in such detail.

Lund et al. [1] evaluated FX activation rates in the presence of high phospholipid concentrations, varying activated FIX levels, and, critically, a limiting concentration (0.1 nM) of each FVIIIa mimetic. This specific setup revealed that the 2 next-generation antibodies use fundamentally different optimization strategies. The zemocimig SIA relies on an exceptionally high and complex assembly affinity (apparent 0.31 nM), acting as a “strong magnet” that efficiently bridges antigens. In stark contrast, denecimig exhibits a lower assembly affinity (16.4 nM) but compensates with an extraordinary specific catalytic activity (29.6 min^-1^), acting as a “high-speed engine” for FXa generation. The presentation of these data is invaluable for defining the unique biochemical profiles of these agents (Figure).

**Figure:**
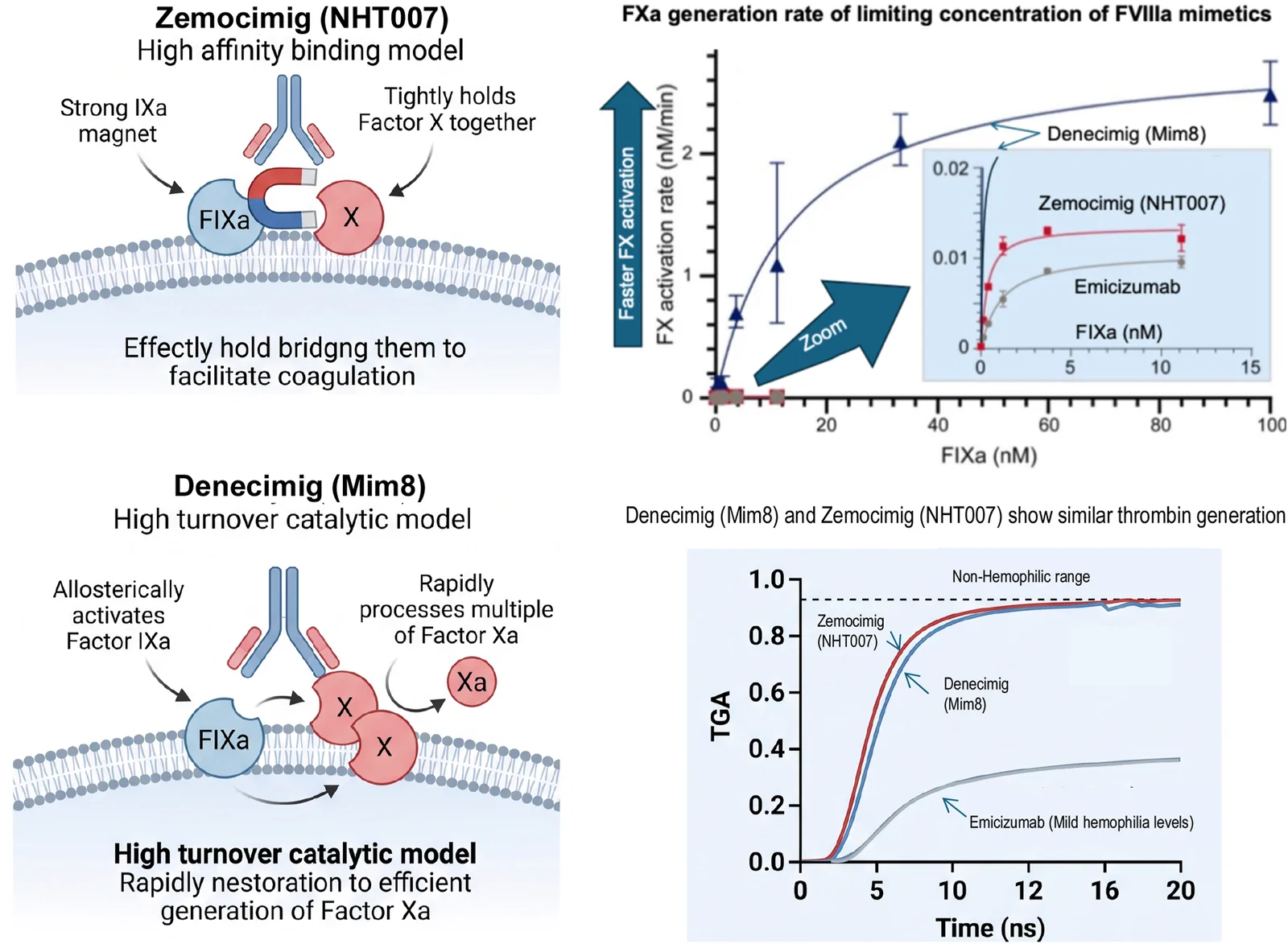
Divergent molecular strategies of next-generation activated factor (F)VIII (FVIIIa) mimetics and their impact on coagulation kinetics. (Left panel: biochemical strategy). Conceptual representation of the distinct optimization paths utilized by next-generation bispecific antibodies. Zemocimig (NXT007) employs an “affinity-driven” strategy, acting as a high-affinity molecular bridge (magnet) that stabilizes the activated FIX (FIXa)-FX complex assembly (0.31 nM). Denecimig (Mim8) uses a “turnover-driven” strategy, functioning as a high-speed catalytic engine that compensates for lower assembly affinity (16.4 nM) with exceptionally high specific activity (29.6 min^-1^). Despite these divergent approaches, both next-generation mimetics successfully overcome the “efficacy ceiling” seen with first-generation agents, restoring thrombin generation potential to the nonhemophilic range. (Right panels: kinetic profile and thrombin generation). Comparative analysis of activated FXa (FXa) generation rates (top) in the presence of limiting concentrations (0.1 nM) of FVIIIa mimetic antibodies and the specrum of thrombin generation (bottom). A consistent color scheme is applied across these functional panels to distinguish the 3 products: zemocimig (NXT007, red line), denecimig (Mim8, dark blue line), and emicizumab (light gray line). The specific experimental concentration (0.1 nM) is significantly lower than therapeutic plasma levels to emphasize the inherent mechanistic differences between the molecules. Despite their divergent molecular strategies, both next-generation mimetics converge on a similar potential to restore thrombin generation to the nonhemophilic range. Data are modified from Lund et al. [1]. TGA, thrombin generation assay.

## 3 Convergence in Thrombin Generation and Clinical Implications

Despite these divergent molecular paths, thrombin generation assays in human HA plasma demonstrated that denecimig and zemocimig SIAs converge on similarly enhanced hemostatic potential. Both agents exhibited comparable concentration-response profiles that clearly exceeded the potency of the emicizumab SIA under standardized conditions.

These findings suggest that both “affinity-driven” and “turnover-driven” strategies can successfully break the previous “ceiling of efficacy” associated with first-generation mimetics, restoring thrombin generation toward the nonhemophilic range. This provides critical insight into the potential of next-generation HA prophylaxis to provide superior clinical protection.

## 4 The Evolving Landscape of Nonfactor Therapies

The treatment of hemophilia is rapidly shifting from traditional intravenous factor replacement to subcutaneous nonfactor therapies that offer improved convenience and steady-state protection [9,10,[16], [17], [18], [19], [20]]. While next-generation FVIIIa mimetics promise to further elevate the standard of care, they also introduce new clinical questions. As we move closer to the “normal range” of coagulation, precision in monitoring and careful management of thrombogenic risks, particularly during breakthrough bleeding or surgery, will become paramount. The biochemical framework provided by Lund et al. [1] will be indispensable for interpreting how these molecular-engineered features translate into real-world safety and efficacy for patients.
